# Mitochondrial biogenesis: An update

**DOI:** 10.1111/jcmm.15194

**Published:** 2020-04-12

**Authors:** Lucia‐Doina Popov

**Affiliations:** ^1^ “Nicolae Simionescu” Institute of Cellular Biology and Pathology of the Romanian Academy Bucharest Romania

**Keywords:** ageing, cancer, metabolic diseases, mtDNA, neurodegeneration, nuclear respiratory factors, PGC‐1α, transcription factor A

## Abstract

In response to the energy demand triggered by developmental signals and environmental stressors, the cells launch the mitochondrial biogenesis process. This is a self‐renewal route, by which new mitochondria are generated from the ones already existing. Recently, considerable progress has been made in deciphering mitochondrial biogenesis‐related proteins and genes that function in health and in pathology‐related circumstances. However, an outlook on the intracellular mechanisms shared by the main players that drive mitochondrial biogenesis machinery is still missing. Here, we provide such a view by focusing on the following issues: (a) the role of mitochondrial biogenesis in homeostasis of the mitochondrial mass and function, (b) the signalling pathways beyond the induction/promotion, stimulation and inhibition of mitochondrial biogenesis and (c) the therapeutic applications aiming the repair and regeneration of defective mitochondrial biogenesis (in ageing, metabolic diseases, neurodegeneration and cancer). The review is concluded by the perspectives of mitochondrial medicine and research.

## 1 MITOCHONDRIAL HOMEOSTASIS

Mitochondria are the major source of energy for the cellular activity, by ATP generation *via* oxidative phosphorylation. Emerging evidence of the last decade indicates that mitochondria form a highly dynamic intracellular network that executes the “quality control” of the organelle's population in a process that implies their fusion, fission and autophagic degradation (known as ‘mitophagy’). Mitochondria regulate the operation of intracellular signalling cascades, generate reactive oxygen species (ROS), execute fatty acids β‐oxidation, participate in aminoacid metabolism, pyridine synthesis, phospholipid modifications, calcium regulation and cells survival, senescence and death. The homeostasis of any healthy cell implies also a controlled regulation of mitochondrial mass and function, as an adaptive response to safeguard the mitochondrial (mt) DNA and to meet the energy demands vital for cellular function.

Mitochondrial homeostasis is preserved by the fine co‐ordination between two opposing processes: generation of new mitochondria, by mitochondrial biogenesis, and the removal of damaged mitochondria, by mitophagy.^1^, ^2^ Among the specific molecules involved in this fine‐tuning, the recent data highlight the peroxisome proliferator‐activated receptor‐γ coactivator (PGC)‐1α, the main regulator of mitochondrial biogenesis,^3^, ^4^, ^5^ the PTEN‐induced putative kinase 1 (PINK1)‐Pakin,^6^ that activates protein synthesis in damaged mitochondria, and the ligand‐activated transcription factor aryl hydrocarbon receptor, that functions also as protector from oxidative stress.^7^

In examining mitochondrial homeostasis, one should consider the particular traits of these organelles in eukaryotic cells: (a) they have a prokaryotic origin and were acquired by eukaryotic cells *via* an endosymbiotic event, (b) are semi‐autonomous organelles: synthesize a rather small number of proteins by transcription and replication of mtDNA‐encoded genes, while the larger proportion of mitochondrial proteome (~60%‐70%^8^ or more than 95%^9^) is nuclear‐encoded, synthesized on cytosolic ribosomes, and finally, sorted and imported to the appropriate intra‐mitochondrial location. The encoding factors for nuclear genes identified so far are as follows: PGC‐1α, the transcription factor A (TFAM), the uncoupling proteins 2 (UCP2) and the uncoupling proteins 3 (UCP3),^10^ (c) mitochondria biogenesis implies a specific route consisting in the recruitment of the novel proteins by the pre‐existing mitochondria, followed by their fragmentation, *via* fission. Associated with the rapid cell growth and proliferation, these events ensure the constant renewal of the mitochondrial population.^6^ Uncovering the complexity of mitochondrial biogenesis operation is an exciting ongoing topic, and its main features are briefly examined next.

## 2 MITOCHONDRIAL BIOGENESIS MACHINERY‐ THE ASSOCIATED SIGNALLING PATHWAYS

**Figure 1:**
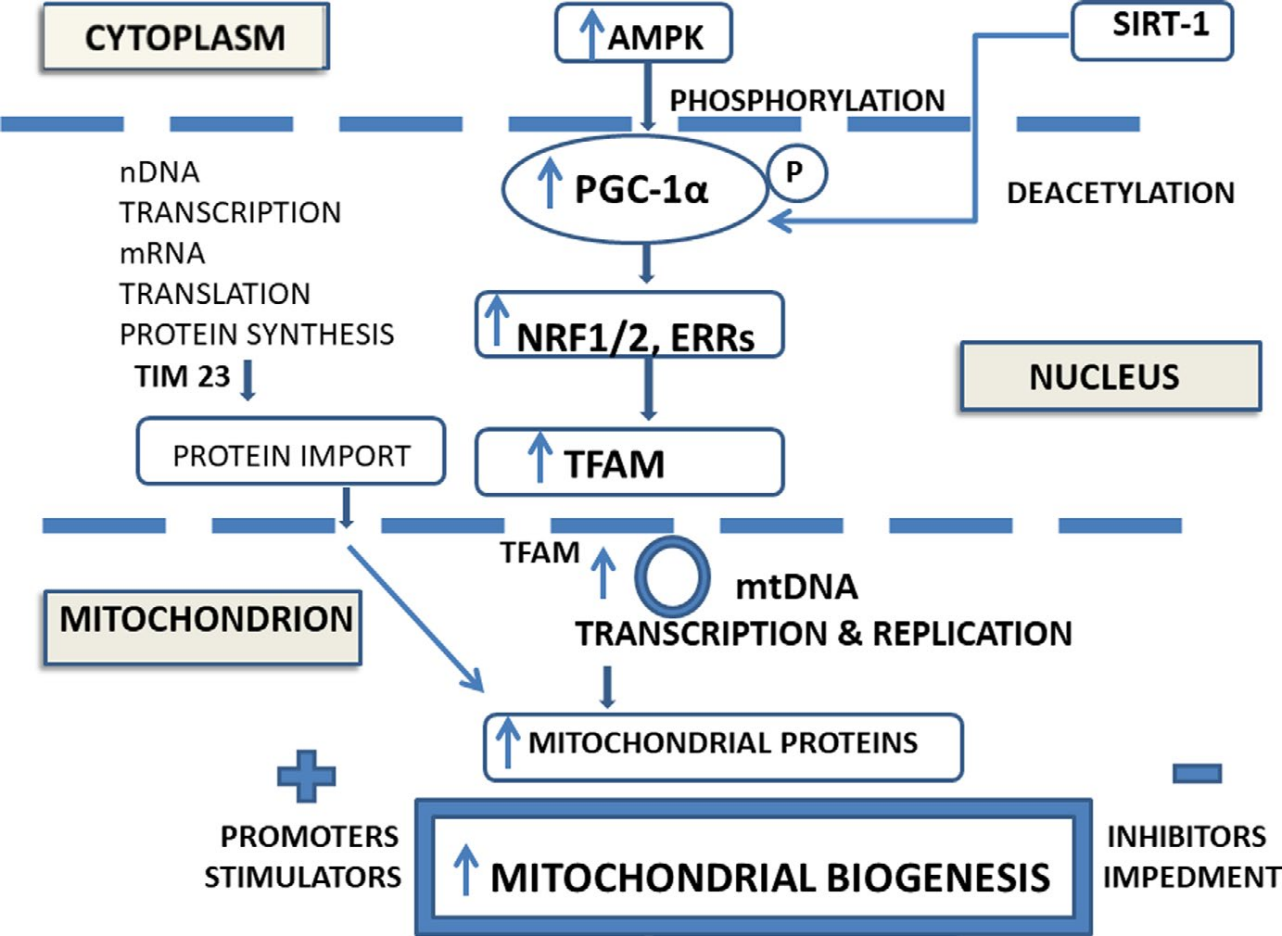
Mitochondrial biogenesis in brief: the central role of PGC‐1α activation and the contribution of proteins encoded by both nuclear and mitochondrial genomes (nDNA and mtDNA) to enhance the mitochondrial proteins content. AMPK, 5' adenosine monophosphate‐activated protein kinase; ERR, the oestrogen‐related receptor; NRF, the nuclear respiratory factor; PGC‐1α, the peroxisome proliferator‐activated receptor‐γ coactivator‐1α; SIRT‐1, the silent information regulator‐1; TFAM, the transcription factor α; TIM 23, translocase

The process of mitochondrial biogenesis takes place mainly in healthy cells. Interesting, in cancerous cells enhanced oxidative phosphorylation and mitochondrial biogenesis were correlated with invasion and metastasis.^11^ It engages co‐ordination between the mitochondrial and the nuclear genomes, in a complex and multistep process (Figure 1) that involves:

(i) **mtDNA transcription and translation** mtDNA transcription is activated by the family of PGC‐1 proteins (PGC‐1α, PGC‐1β and PGC‐1), from which PGC‐1α is considered the *master regulator* of mitochondrial biogenesis. The pathway is initiated by PGC‐1α activation (by either phosphorylation or deacetylation), followed by stimulation of a series of nuclear transcription factors, that is the nuclear respiratory factor‐1(NRF‐1), NRF‐2 and oestrogen‐related receptor‐α (ERR‐α), and by the increase in expression of TFAM, the final effector of mtDNA transcription and replication.^12^, ^13^, ^14^ Next, translation of the mtDNA‐encoded genes into proteins takes place with the assistance of specific translation factors (encoded by nuclear DNA, nDNA), such as the initiation factor 2 and 3 (mtIF2 and mtIF3), the elongation factors Tu, Ts and G1 (mtEFTu, mtEFTs and mtEFG1), the translational release factor1‐like (mtRF1L) and the recycling factors (mtRRF1 and mtRRF2); furthermore, the levels of mitochondrial proteins are regulated by the translational activator of cytochrome c oxidase 1 (TACO1) that binds the mitochondrial RNA (mRNA).^15^

(ii) **Synthesis, import and assembly of mitochondrial proteins encoded by nDNA** These mitochondrial proteins originate from the preproteins synthesized within the cytosol and provided with an amino‐terminal cleavable targeting signal. The translocase TIM23 directs the signal of preproteins towards the mitochondrial matrix, where they assemble, and are sorted to a precise intra‐mitochondrial location, that is the matrix or the inner mitochondrial membrane (IMM). The energy required for driving this import pathway is provided by the mitochondrial membrane potential and the ATP (by oxidative phosphorylation).^8^, ^16^ The biogenesis of the outer mitochondrial membrane (OMM) proteins has been studied so far in unicellular organisms, such as the yeast *Saccharomyces cerevisiae*; the OMM functions as an interface with the cytosol and is particularly important for mitochondrial dynamic changes (fission, fusion) and interaction with other intracellular organelles.^9^
  The mitochondrial biogenesis markers are the mtDNA copy numbers, the elevated mtDNA:nDNA ratio and the level of mitochondrial gene expression.^1^, ^17^ In cancer cells,^18^ an augmented expression of PGC‐1α,^19^ NRF1 and TFAM was reported, although these cells have a reduced number of mitochondria.^20^ Moreover, a recent report underlines that the use of TFAM level as a biogenesis marker is questionable, as it does not always match the mtDNA copy number, and the expression of mtDNA‐encoded polypeptides.^21^
  What are the consequences of mitochondrial biogenesis? The current data acknowledge the increase in the oxidative phosphorylation capacity, the diminishment of pathologic oxidative stress and the repair of mitochondrial‐associated dysfunctions.^13^
  How can be measured mitochondrial biogenesis? Reliable strategies are based on the magnitude of mtDNA synthesis and of mitochondrial membrane phospholipids. In this context, two cautions are noted: (a) a change in the number of mitochondria is not indicative of biogenesis, as their amount is not exclusively due to synthesis,^22^ and (b) mitochondrial biogenesis may conduct to detrimental effects, such as the import of misfolded proteins into the organelle, and the silencing of the unfolded protein response in the endoplasmic reticulum.^23^

### 2.1 Mitochondrial biogenesis inductors/promoters

Mitochondrial biogenesis induction is associated with *activation of transcription factors* that act on mitochondrial genes and with *up‐regulation of local translation of mitochondrial proteins*. These effects are produced in response to several natural products, such as 6‐gingerol (the main active component of the ginger extracts)^24^ and Ursolic acid (a natural triterpene).^25^ In contradistinction, relatively few synthetic drugs have been identified as mitochondrial biogenesis inductors.^12^

Reportedly, the following signalling pathways sustain *transcription activation* during mitochondrial biogenesis:

- the AMPK/ PGC‐1α pathway used by C1q/tumour necrosis factor‐related protein‐3 (CTRP3) to promote biogenesis in cardiomyocytes,^26^ and by the ginger extract, in both mice and HepG2 cells.^24^ Furthermore, AMPK phosphorylates and activates histone acetyltransferase 1 (HAT1), creating a more relaxed chromatin‐DNA structure that favours transcription; AMPK phosphorylates also the epigenetic factor DNA methyltransferase 1 (DNMT1) that limits transcription factors access to promoters.^10^ A recent report shows that the diterpene alkaloid benzoylaconine activates AMPK signalling cascade and stimulates mitochondrial biogenesis^27^;
- the induction of PGC‐1α along with its downstream target genes, NRF1 and TFAM. Such signalling cascade was identified in pancreatic MIN6 β‐cells, after the humanin treatment,^28^ and in 3T3‐L1 pre‐adipocytes, after salicylate medication.^29^ Activation of PGC‐1α signalling pathway is mediated also by the transcription factor cAMP response element‐binding protein (CREB); it binds to certain DNA sequences (the cAMP response elements) and subsequently increases/decreases genes transcription. In endothelial cells, CREB/ PGC‐1α pathway promotes mitochondrial biogenesis by activation of G protein‐coupled receptor (TGR5),^30^ the route operating also after lixisenatide medication, a drug approved by the US Food and Drug Administration for the treatment of type 2 diabetes^31^;
- stimulation of the Gβγ (a component of heterotrimeric G proteins)‐Akt‐eNOS‐sGC (soluble guanylatecyclase) pathway by the β2 adrenergic receptor agonists, such as formoterol, allowing recovery from acute and chronic degenerative diseases,^13^ and carvedilol, employed in heart failure^32^;
- the return to normal of the Akt/ transcription factor FoxO3a axis under the influence of dietary β‐hydroxy‐β‐methylbutyrate (HMB), is another condition that improves mitochondrial biogenesis^33^;
- the sirtuins assistance in transcription: it is known that the silent information regulator‐1 (SIRT1) activates the PGC‐1α‐mediated transcription of nuclear and mitochondrial genes encoding for proteins during mitochondria proliferation, oxidative phosphorylation and energy production, while SIRT3 stimulates the proteins important for oxidative phosphorylation, tricarboxylic acid cycle and fatty‐acid oxidation, and indirectly, the PGC‐1α and AMPK.

Taken together the above data, it is obvious that up‐regulation of transcription factors is a key event in mitochondrial biogenesis. However, depending on ligands specificity, unwanted genes may be equally activated, conducting to detrimental (neurological and hyperproliferative) effects.^12^

Up‐regulation of *mitochondrial proteins translation* is associated with exercise‐induced mitochondrial biogenesis (in the *plantaris* muscle).^15^ An interesting mechanism implied in biogenesis of healthy mitochondria was deciphered in *Drosphila*: the MDI protein of the mitochondrial OM recruits the translational stimulator La‐related protein (Larp) and promotes the synthesis (on mitochondrial surface) of a subset of nuclear‐encoded mitochondrial proteins by cytosolic ribosomes.^6^

### 2.2 Mitochondrial biogenesis stimulators and inhibitors

In physiological conditions, the response of cells to energy demands leads to either up‐ or down‐regulation of the transcription factors that stimulate and/or inhibit mitochondrial biogenesis, respectively. The pathology‐associated disturbances of mitochondrial biogenesis consist in an impeded mitochondrial biogenesis, a condition in which stimulation of the declined process is required, or in abnormal higher levels of this process, when and a diminishment is necessary.

Examples of efficient *stimulators of mitochondrial biogenesis* are the followings: formoterol, used for treating podocytopathies,^18^ resveratrol (a polyphenol), that prevents rotenone‐induced neuronal degeneration,^34^ acetylcholine, protector in hypoxia/reoxygenation injury,^35^ adiponectin, a cardioprotector in diabetes,^36^ and tetrahydrobiopterin, helpful for the cardiac contractility.^37^ The cellular mechanism beyond the above stimulatory effects on mitochondrial biogenesis entails the up‐regulated expression of the transcriptional regulator PGC‐1α. Recently, normalization of Akt/FoxO3 axis was reported to be involved in the protective effects of dietary HMB against lipopolysaccharide (LPS)‐induced muscle atrophy.^33^ Another regulatory mechanism is based on phosphorylation of GSK‐3β exerted by arachidonyl‐2‐chloroethylamide (ACEA, a selective agonist of cannabinoid receptor1) effective at the beginning of cerebral ischemia.^38^

Several natural extracts have been found to stimulate mitochondrial biogenesis. Such regulatory effects were recently reported for the *Kaempferia parviflora* extracts (containing methoxyflavones), that act through the SIRT1/AMPK/PGC‐1α/PPARδ pathway,^39^ for tangeretin (a polymethoxylated flavonoid of mandarin fruits), activator of AMPK/PGC‐1α pathway,^40^ for salidroside (isolated from *Rhodiola rosea L.*), that stimulates the miR22/SIRT1 pathway,^41^ for the spice saffron (*Crocus Sativus L.*), that augmented NRF‐1 gene expression in exercised rats,^42^ and for the natural precursor of resveratrol, polydatin that enhances SIRT1 expression.^43^

*The inhibitors of mitochondrial biogenesis* down‐regulate the expression of the associated‐transcription factors, such as PGC‐1α, TFAM and AMPK. The activity of PGC‐1α pathway is reduced by miR‐130b‐p,^44^ 2‐methoxyestradiol,^45^ cyclosporine A,^46^ XCT790 (a potent and selective inhibitor of the oestrogen‐related receptor α)^47^ and the high glucose high‐fat environment.^36^ The down‐regulation of TFAM takes place at the use of the local anaesthetic ropivacaine^48^ and at the in vitro treatment of cells with silica nanoparticles.^49^ Furthermore, the diminished AMPK expression explains resistin inhibitory effects on mitochondrial biogenesis.^50^

It is evident that reduced biogenesis is accompanied by other mitochondrial dysfunctions, such as an impaired ATP synthesis capacity leading to acceleration of mitophagy, critical for cell health. A reduced mtDNA/ nuclear ratio^1^ has also been reported to be associated with the impairment of biogenesis/altered biogenesis.

The examination of the two opposite sides of mitochondrial biogenesis, that is the impairment (such as in ageing, metabolic and neurodegenerative diseases) and the abnormal intensification (in some tumours) conducted in the last decade to identification of several strategies adequate for exploitation in therapy. Examples are discussed next.

## 3 DYSREGULATION OF MITOCHONDRIAL BIOGENESIS; REPAIR STRATEGIES

### 3.1 Ageing

The cells senescence and the consequent ageing is associated with the impairment of mitochondrial biogenesis and bioenergetic potential, the decrease in mitochondrial dynamics, the altered quality control, the failure in mtDNA repair, the accumulation of mtDNA mutations and the decline in mitophagy.^51^, ^52^, ^53^, ^54^ The main factors involved in ageing effects on mitochondrial biogenesis are the reduced activity of AMPKα and the decreased expression of SIRT1, PGC‐1α, TFAM and NRF‐1,2,^55^, ^56^ along with the regulatory loop that engages PGC‐1α and NRF‐2 interaction.^57^ Strategies to prevent/delay age‐associated decline in mitochondrial biogenesis consists in stimulation of PGC‐1α signalling with tetrahydrobiopterin^37^ or with resveratrol,^58^ modulation of TFAM binding to mtDNA,^59^ mitophagy regulation,^60^ dietary supplementation with acetyl‐l‐carnitine (ALCAR),^51^, ^53^ cells exposure to gomisin A (a bio‐active compound isolated from the fruit of *Schisandra chinensis*),^61^ the regular exercise training, and the calorie restriction.^51^ The current endeavours aimed to delay/counteract the age‐associated decline of mitochondrial biogenesis may have translational relevance for promotion of a healthy ageing, for protection against age‐related pathologies and for the improvement of the quality of life of the elderly.

### 3.2 Metabolic diseases

The impairment of mitochondrial biogenesis and function has been linked to metabolic diseases such as type 2 diabetes and obesity. In diabetic kidney, the mechanism beyond the reduced mitochondrial biogenesis implies the decrease of PGC‐1α/AMPK/SIRT‐1 signalling pathway.^62^ In placentae of diabetic mothers, impaired mitochondrial biogenesis engages PGC‐1α/TFAM signalling pathway and is mainly present at male offspring; this trait may explain the propensity for development of future metabolic diseases in adult males.^63^ In diabetic heart, earlier studies reported that hypoadiponectinemia impaired AMPK‐PGC‐1α signalling^64^; more recently, in a model for type 2 diabetes (a high glucose/high‐fat medium) adiponectin was found to partial rescue mitochondrial biogenesis in cardiomyocytes, *via* PGC‐1α‐mediated signalling.^36^ This pathway participates in cardioprotection and is evaluated as a novel therapeutic target.^65^

Mitochondrion is regarded now as a possible target for the prevention and treatment of chronic metabolic disorders; in this context, the endurance exercise it is routinely used to alleviate the reduced mitochondrial biogenesis.^66^ Furthermore, the antidiabetic effect of mitochondrial biogenesis enhancers, such as *Spirulina platensis* ^67^ and Alogliptin (a dipeptidyl‐peptidase‐4 inhibitor)^68^ were recently reported.

Another ongoing topic is the regulation of mitochondrial biogenesis in adipocytes.^69^, ^70^ The obesity‐associated signalling entails hyperacetylation of PGC‐1α,^71^ and induction of pAMPK, PGC‐1α, NRF‐1 and TFAM (after the salicylate treatment of pre‐adipocytes).^29^ Activation of AMPK along with stimulation of mitochondrial gene expression and mtDNA replication explain the beneficial effects of isorhamnetin (3‐O‐methyl quercetin) on adipocytes mitochondrial biogenesis.^72^ AMPK activation contributes also to the anti‐obesity effects of zeaxanthin (an oxygenated carotenoid) that promotes mitochondrial biogenesis and expression of brown and beige adipogenesis markers.^73^ The regulation of mitochondrial biogenesis in beige adipocytes (in the course of browning) involves PGC‐1α signalling, associated with miR‐494‐3p expression.^74^ Other stimulatory factors of mitochondrial biogenesis are NRF‐1 and the mitochondrial transcription factor A that intervene in metformin effect on brown adipocytes.^75^ In contradistinction, decreased UCP1 expression explains the reduced mitochondrial biogenesis generated by arsenite in brown adipocytes.^76^

The above basic findings may be used as a basis for further clinical approaches in metabolic diseases.

### 3.3 Neurodegeneration

Mitochondrial biogenesis is a potential novel therapeutic target for neurodegenerative diseases treatment including Alzheimer's disease (AD), Parkinson's disease (PD), Huntington's disease (HD) and amyotrophic lateral sclerosis (ALS).^1^ Although this strategy is based on a plethora of basic and (pre)clinical results, in the present overview only the data on the intracellular pathways beyond mitochondrial biogenesis are mentioned.

In AD and PD, mitochondrial biogenesis is impaired^77^ and augmenting this process turned into a therapeutic approach. The intracellular mechanism was uncovered in hippocampal neurons, where amyloid β25‐35 inhibits AMPK‐SIRT‐1, PGC‐1α pathway.^78^ Recent reports indicate melatonin, as a promoter of mitochondrial biogenesis,^77^ along with resveratrol, that induced PGC‐1α and mtTFA expression,^34^ berberine (a natural AMPK activator), that stimulates PGC‐1α and NRF‐2 in neuronal cells,^79^ and rotenone, an inhibitor of Complex I.^80^ Moreover, necdin (a melanoma antigen) prevents mitochondria‐associated neurodegeneration by binding to PGC‐1α and suppressing its proteolytic degradation in the ubiquitin‐proteasomal system.^81^, ^82^ Interestingly, mtDNA replication appears to be an early response to neurodegeneration‐associated stress and a precursor for mitochondrial biogenesis in axons.^80^

Distinctly, the neurotoxic effect of some medicines is accompanied by reduced mitochondrial biogenesis. An example is the local anaesthetic ropivacaine (employed in medical and dental care) that reduces expression of mitochondrial regulators PGC‐1α, NRF‐1 and TFAM.^48^

### 3.4 Cancer

It is known that mitochondrial biogenesis targeted therapies are efficient for the prevention and treatment of relapsed and resistant cancers.^47^ The pointed intracellular pathways are PGC‐1α (important also for the cells adaptive response against chemotherapeutic stress),^83^ AMPK (a proximal signalling step for mitochondrial biogenesis)^84^ and dynamin‐related protein‐1 (Drp1) up‐regulation, accompanied by augmented levels of PGC‐1α, NRF‐1 and TFAM.^19^ Among the modulators of mitochondrial biogenesis, sulforaphane (a sulphur‐rich compound found in cruciferous vegetables) is considered a potential antineoplastic agent; in prostate cancer cells, it stabilizes NRF‐2, increases the expression of PGC‐1α and decreases the level of hypoxia‐inducible factor‐1α (HIF‐1α).^85^ Chemotherapy medication with cisplatin stimulates PGC‐1α expression and up‐regulates mitochondrial biogenesis.^83^

Mitochondrial biogenesis is increased in some invasive cancer cells, such as osteosarcoma; the use of 2‐methoxyestradiol inhibits biogenesis, *via* regulation of PGC‐1α, COX1 and SIRT‐3.^45^ In this circumstance, the strategy to stop the increased propagation of cancer stem cells consists in doxycycline inhibition of mitochondrial biogenesis.^86^

## 4 CONCLUSION AND PERSPECTIVES

The mitochondrial biogenesis is a complex biological process (Figure 1), that controls organelle's self‐renewal and the maintenance of mtDNA, ensuing cell homeostasis. This topic is under intense investigation at present. The intracellular signalling pathways uncovered so far identified PGC‐1α as a master regulator of mitochondrial biogenesis, implicated in the response to several inductors/promoters, stimulators and inhibitors. Dysregulated mitochondrial biogenesis occurs not only in senescence and ageing, but also in metabolic diseases, neurodegeneration and cancer, and is potentially ameliorated by the novel mitochondria‐based therapies. However, there are still several issues that require an answer, such as the association between impaired mitochondrial biogenesis and the early stage of myocardial remodeling,^87^ the neuron‐specific mechanism of mitochondrial biogenesis,^81^ and the limitation of osteoarthrosis progression in chondrocytes,^55^ among others. Challenging topics are the exploitation of mitochondria‐based therapies for the treatment of chronic degenerative diseases^12^, ^13^ and for the cancer prevention.^88^

## CONFLICT OF INTEREST

The author confirms that there is no conflict of interest.

## Data Availability

Most of the 88 references are associated with the corresponding DOI reference number.
